# Deterioration of traditional dietary custom increases the risk of lifestyle-related diseases in young male Africans

**DOI:** 10.1186/1423-0127-17-S1-S34

**Published:** 2010-08-24

**Authors:** Atsumi Hamada, Mari Mori, Hideki Mori, Alfa Muhihi, Marina Njelekela, Zablon Masesa, Jacob Mtabaji, Yukio Yamori

**Affiliations:** 1 Institute for World Health Development, Mukogawa Women's University, Nishinomiya, Hyogo, 663 8143, Japan; 2 Department of Physiology, Muhimbili University of Health and Allied Sciences, P. O. Box 65001, Dar es Salaam, Tanzania; 3 Department of Physiology, Weill Bugando University College of Health P. O. Box 1464, Mwanza, Tanzania

## Abstract

**Background:**

Prevalence of metabolic syndrome (MS) is rapidly increasing worldwide. To investigate the spread of MS risks and its relationship with eating habits including fish intake, we carried out a health examination for young and middle-aged men.

**Methods:**

The subjects were 97 healthy men (20 to 50 years) living in Mwanza, located on the shore of Lake Victoria in Tanzania. The health examination was conducted according to the basic protocol of WHO-CARDIAC (Cardiovascular Diseases and Alimentary Comparison) Study. This survey included anthropometric measurements, a dietary questionnaire, blood pressure measurement, and blood and 24-hour urine (24U) collection. Excretions of sodium, potassium and taurine (Tau) in 24U were estimated as the biomarkers of salt, vegetable and fish product intakes respectively.

**Results:**

In this survey, 62.5 % of the young and 63.3% of the middle-aged adults had MS risks. The most prevalent MS risk factor was increased blood pressure (50.0% of young adults and 53.1% of the middle-aged). Tau excretions in 24U and n-3 fatty acid levels in plasma were significantly lower in young adults than those in the middle-aged (both *P* < 0.05). The eating frequencies of non-traditional foods such as donuts and ice cream showed negative correlations with age (r = -0.282, *P* < 0.01 and r = -0.246, *P* < 0.05), while salt intake positively correlated with age (r = 0.236, *P* < 0.05). Tau excretion in 24U was inversely correlated with atherosclerosis index (r = -0.306, *P* < 0.01) and fasting blood glucose (r = -0.284, *P* < 0.05).

**Conclusions:**

Young adults in Mwanza had a decreased frequency of eating habit of fish products compared with the middle-aged as indicated by Tau excretion in 24U and n-3 fatty acid level in the plasma, and over half of young adults had one or more MS risks just as the middle-aged. The change in food habit of lowered fish intake and raised exotic food intake might be concluded to increase MS risks in young men.

## Background

Metabolic syndrome (MS) is an increasing health problem throughout the world. Its epidemic is a severe problem among the elderly and young adults as well [1]. People suffering from MS, such as obesity, high blood pressure (BP), low level of high-density lipoprotein cholesterol (HDL-C), high level of triglycerides (TG) and impaired fasting blood glucose (FBG), are more susceptible to cardiovascular diseases (CVD) and diabetes. Even in developing countries CVD have become a major health problem and a leading cause of death [2,3]. MS (or CVD and diabetes) is likely to become a serious public health problem in the future. To prevent such an epidemic, it is important to evaluate and identify MS risks in young adults.

In the WHO-Cardiovascular Diseases Alimentary Comparison (CARDIAC) Study the relation of CVD mortalities with biological markers of diet was assessed using 24-hour urine (24U) analysis; a more reliable method for dietary habit assessment than questionnaire [4]. Taurine (Tau) excretion in 24U was inversely associated with age-adjusted mortality rates of CVD in CARDIAC Study [5,6]. It was shown that the excretions of Tau, sodium (Na) and potassium (K) in 24U correlated well with dietary fish, salt and vegetable intakes, respectively, which credibly linked to the MS risks [7]. Tau is found in many foods but is most abundant in seafood. Recent epidemiological studies and animal experiments have shown that a high-fish diet, or intake of Tau, prevents MS risks including obesity, hypertension and hypercholesterolemia. Thus, Tau excretion might be a useful indicator for assessment of potential MS risks [8-10].

Our previous study in Tanzania showed that the prevalence of hypertension and obesity has been increasing in urban areas more than in rural areas (aged 48-56 years) [11]. A cross sectional population study of Tanzanian people living in three differential (urban, rural and semi-nomadic) areas showed that the frequency of fish intake was inversely associated with CVD risks [12]. In this study, we conducted a health survey to assess MS risks in young and middle-aged male Africans living in the lakeshore area, an area that has not been investigated before. Furthermore, we evaluated the potential relationship between dietary habit and MS risks in young male Africans as compared to those of middle-aged males.

## Methods

### Subjects

We carried out a health survey in urban Mwanza, the second largest city in Tanzania located on the shore of Lake Victoria. Participants were 97 men aged 20 to 50 years who were informed of the purpose and procedures about the study and signed an informed consent form. The study included a BP survey, anthropometrical measurements, blood and 24U collection and a lifestyle questionnaire. This study was approved by the Ethics Committees of Mukogawa Women’s University and Weill Bugando University College of Health.

### Data collection

All measurements and blood sampling were performed by an experienced physician and a nurse at a local community healthcare centre or hospital. Body weight and height were measured with subjects standing and wearing light clothes. From these results, body mass index (BMI; weight (kg) / height (m)^2^) was calculated. Waist circumference (WC) was measured using a flexible steel measuring tape. Blood pressures and pulse rates were measured after a 5- to 10-minute rest using an automatic digital BP measurement system (Omron Digital HEM-907, Tokyo, Japan). The mean of 3 readings was used in this analysis.

Blood samples were taken after at least 10 hours of fasting. Blood analyses including serum total cholesterol (TC), HDL-C, TG, FBG, haemoglobin A1c (HbA1c), eicosapentaenoic acid (EPA) and docosahexaenoic acid (DHA) were assessed at SRL Inc. (Tokyo, Japan). Enzymatic methods for TC, HDL-C, TG and FBG, a latex agglutination method for HbA1c and a gas chromatographic method for EPA and DHA were performed. Non-HDL-C was calculated by subtracting HDL-C from TC. The atherosclerosis index (AI) was calculated as non-HDL-C / HDL-C.

24U was collected using an aliquot cup (Izumi Seisakusho, Osaka, Japan) and various markers of diets were analyzed, including creatinine, Na, K and Tau. Na and K were determined by electrode methods and creatinine was analyzed by enzymatic method. Tau was assessed using high performance liquid chromatography. Sodium-potassium ratio (Na/K) was calculated by the measured values and the amounts of daily excretion for Tau and sodium chloride (NaCl) were also calculated by the 24U volume. Success or failure in 24U collection was judged from the diagnostic criteria based on calculated values of the daily creatinine excretion in 24U. The results of those who failed to complete 24U collection were excluded from the analyses.

We used International Diabetes Federation (IDF) criteria to define the MS risks: WC ≥ 94 cm, BP ≥ 130/85 mmHg, FBG levels ≥ 100 mg/dl (5.6 mmol/l), TG levels ≥ 150 mg/dl (1.7 mmol/l) and HDL-C levels < 40 mg/dl (1.0 mmol/l) in men. Since the criteria was set to be lower than other criteria such as in the WHO or the National Cholesterol Education Program-Adult Treatment Panel Ⅲ definitions, we could detect people having MS risks at an earlier stage.

Intake frequency was assessed using a food frequency questionnaire about 135 food items. For each, eating frequency was assessed and categorized into 9 levels; never in a month, 1 to 3 times per month, once per week, 2 to 4 times per week, 5 to 6 times per week, once per day, 2 to 3 times per day, 4 to 5 times per day and 6 or more times per day. For simplification in some cases for analyses, they were divided into 4 groups; less than once per month, 2 to 4 times per week, 5 to 6 times per week and more than once per day. Furthermore, information regarding employment, education, alcohol intake, smoking, medical history and physical activity were recorded.

### Data analysis

Differences between young adults and the middle-aged were investigated using the Student’s *t* test. Prevalence rates of MS risk factors were compared using a chi-square test. Relationships between two parameters were assessed by a calculation of Pearson’s correlation coefficients. To compare characteristics among the three groups classified by the excretion levels of Tau in 24U, analyses of one-way variance was used. All statistical analyses were undertaken using the SPSS for windows package version 15 (SPSS Inc, Chicago, IL). Results are presented as means ± standard deviations. A *P* value of 0.05 was set as the level of significance.

## Results

### Characteristics of the young and the middle-aged adults

A total of 97 men participated in this study and 74 men completed 24U collections successfully (76.3% of all). The average age was 31.2±6.4 for all participants and 31.5±6.2 years old for those who completed 24U, respectively. The characteristics of young and middle-aged participants are shown in Table 1. The excretions of Tau in 24U and DHA levels in plasma were significantly higher in the middle-aged than in young adults (both *P* < 0.05). In contrast, Na/K ratio was markedly lower in the former than in the latter (*P* < 0.01). Waist circumference was markedly higher among the middle-aged than young adults (*P* < 0.05). BP, FBG and cholesterol levels did not vary significantly between the generation groups. Comparing the prevalence of MS risk factors such as increased WC, increased BP, elevated FBG, lowered HDL-C, and increased TG, no significant difference was observed between young and middle-aged men (Table 2). Almost half of participants showed high BP; 50.0% in young adults and 53.1% in the middle-aged and the number of people with impaired FBG was especially low.

**Table 1:** Characteristics of study subjects

		Young	Middle-aged	*P* value*
		≤30 (n=36)	≥31 (n=38)	
	Age (year)	26.4±2.8	36.2±4.5	-

Anthropometric measurements				

	BMI (kg/m^2^)	21.8±2.2	22.6±3.1	0.47
	WC (cm)	75.3±4.2	79.7±8.6	<0.05
	SBP (mmHg)	132.1±11.9	129.6±17.2	0.47
	DBP (mmHg)	69.3±9.3	70.8±12.8	0.56

Blood analysis				

	FBG (mg/dl)	78.1±8.7	75.5±9.2	0.22
	HbA1c (%)	4.99±0.40	5.00±0.65	0.90
	TC (mg/dl)	154.5±34.3	156.5±37.1	0.81
	HDL-C (mg/dl)	46.0±9.0	52.3±19.0	0.14
	non-HDL-C (mg/dl)	108.6±34.2	104.2±30.0	0.57
	TG (mg/dl)	76.4±45.5	76.5±39.5	0.99
	AI	2.47±0.95	2.16±0.75	0.16
	EPA (µg/ml)	19.5±11.9	26.7±22.3	0.09
	DHA (µg/ml)	75.2±20.8	90.1±36.5	<0.05

Urine analysis				

	NaCl (g/day)	6.10±3.11	5.83±2.74	0.69
	K (g/day)	1.18±0.42	1.62±0.77	<0.01
	Na/K	3.77±2.48	2.59±1.15	<0.01
	Taurine (µmol/day)	729.6±499.3	998.5±587.1	<0.05

*For difference between the young and the middle-aged adults by Student’s *t* tests. Data are expressed as mean ± SD.

**Table 2:** Prevalence of MS risks

	Young	Middle-aged
	% (n)	% (n)
WC (≥ 94 cm)	2.1 (1)	10.2 (5)
BP (≥130/85 mmHg)	50.0 (24)	53.1 (26)
SBP (≥ 130 mmHg)	50.0 (24)	53.1 (26)
DBP (≥ 85 mmHg)	0 (0)	12.2 (6)
FBG (≥ 100 mg/dl)	2.1 (1)	2.0 (1)
HDL-C (< 40 mg/dl)	20.1 (10)	18.4 (9)
TG (≥ 150 mg/dl)	6.3 (3)	4.1 (2)
		
At least one risk factor	62.5 (30)	63.3 (31)
One risk factor	52.1 (25)	44.9 (22)
Two risk factors	6.3 (3)	12.2 (6)
Cluster	4.2 (2)	6.1 (3)

### Food frequency analysis

The frequency of fish intake was investigated in four types of fish; fried fish, fresh fish, sardine/dagaa and dried fish. As the Tau excretion in 24U of the middle-aged was higher than that of young adults, the frequency of fresh fish intake showed positive correlation with the Tau excretion (data not shown). We analyzed the relationship between intake frequencies of 38 items with age, which were selected limited to identifiable items as good or bad for health. Table 3 gives correlation coefficients, only confined to the items having significant connections. Negative correlation was revealed in six food items, showing the highest correlation of donuts with age (r = -0.282, *P* < 0.01) and otherwise canned juice (r = 0.247, *P* < 0.05) and salt (r = 0.236, *P* < 0.05) identified to be positive correlations with age.

**Table 3:** The correlations between age and food intake frequency

	r	*P* value
Carrot (in salad)	-0.206	<0.05
Irish potato (chips)	-0.203	<0.05
Beef	-0.251	<0.05
Donut	-0.282	<0.01
Ice cream	-0.246	<0.05
Coconut milk	-0.226	<0.05
Canned juice	0.247	<0.05
Salt	0.236	<0.05

The correlations between age and eating frequencies of 38 items were investigated; Donut, Rice cake, Biscuit, Cake, Spinach, Cow pea leaves, Eggplant, Cassava leaves, Cabbage, Chinese cabbage, Pumpkin, Tomato (in salad), Tomato (not in salad), Carrot (in salad), Carrot (not in salad), Beef, Goat, Pork, Liver, Chicken, Fried fish, Fresh fish, Sardines/Dagaa, Dried fish, Eggs, Margarine on bread, Soda, Ice cream, Local brew, Beer, Soya drink, Canned juice, Irish potato(chips), Coconut milk, Vegetable oil, Palm oil, Sugar, Salt. Only significant data are shown (*P* < 0.05).

### The MS risk factors and the excretion levels of Tau in 24-h urine

The associations of urinary level of Tau with MS risk factors were listed in Table 4. The excretion of Tau showed notably positive correlations with EPA (r = 0.506, *P* < 0.001), DHA (r = 0.551, *P* < 0.001) and K (r = 0.320, *P* < 0.01). Inverse correlations were found between the excretion of Tau and AI (r = -0.306, *P* < 0.01), FBG (r = -0.284, *P* < 0.05) and non-HDL-C (r = -0.278, *P* < 0.05). Participants in this analysis were divided into three groups by the excretion levels of Tau (low; under 500 µmol/day, medium; over 500 but less than 1000 µmol/day, high; over 1000 µmol/day, data not shown). There was no significant difference in the numbers of participants among three groups (low, 29.7%; medium, 36.5%; high, 33.8%). DHA level in the plasma of the high Tau group was higher than any other group (*P* < 0.01). The non-HDL-C level in the plasma of the low Tau group was found to have the highest level as compared to three groups (*P* < 0.05). The prevalence of MS risks in the three groups was compared. A significantly high prevalence of increased TG level was observed in the low Tau group compared with the other groups (low, 18.2%; medium, 0%; high, 4.0%; *P* < 0.05, data not shown).

**Table 4:** The correlation coefficients of Tau in 24U with diet-related factors

	r	*P* value
Age	0.189	0.11
BMI	-0.049	0.68
WC	-0.017	0.88
SBP	0.206	0.08
DBP	0.190	0.11
		
FBG	-0.284	<0.05
HbA1c	-0.045	0.70
TC	-0.177	0.13
HDL-C	0.170	0.15
non-HDL-C	-0.278	<0.05
TG	-0.123	0.30
AI	-0.306	<0.01
EPA	0.506	<0.001
DHA	0.551	<0.001
		
NaCl	0.193	0.10
K	0.320	<0.01
Na/K	-0.125	0.29

## Discussion

This study revealed a lower eating level of seafood in young adults than in the middle-aged in Mwanza using the excretion level of Tau in 24U and omega-3 fatty acid levels such as DHA in plasma as bio-markers. Previous studies have reported that the frequency of fish eating is positively related with Tau excretion in 24U justifying its use as a bio-marker of fish intake [13]. Similarly, plasma levels of DHA and EPA have also been shown to be positively correlated with the frequency of fish intake [14]. This study verifies the positive relationship between Tau in 24U with DHA in plasma, and Tau with fresh-fish intake frequency.

It is noteworthy that the prevalence of the MS risk factors in young adults (mean age, 26.4 years) and the middle-aged (mean age, 36.2 years) showed no significant difference. MS risks are known to be more prevalent with advancing age among Africans or Black Americans [15]. The relatively high prevalence of MS risks (62.5%) at this early age implies the exposure to a high risk of cardiovascular disease in future. One of the reasons for this phenomenon might lie on their habitual assimilating of non-traditional foods. The negative correlations of age with the intake frequency of donuts and ice cream indicate a tendency of younger men introducing westernized foods to their diet. In this study, the significant correlations between the eating frequency of such unhealthy foods and socio-economic status including income or education level have not been shown. However, undesirable changes in lifestyle based on urbanization and the socio-economic growth have resulted in the dramatic increase in MS prevalence [16]. A recent study performed in urban Tanzania revealed that the prevalence of obesity increased significantly in 1998 compared to in 1987 [17], and the prevalence of hypertension and obesity has been increasing more in urban areas than in rural areas [11]. A study in Benin, West Africa, revealed that a longer exposure to the urban environment was associated with a higher risk of hypertension and that socioeconomic status was positively correlated with abdominal obesity [18].

Another contribution to the higher risks of young adults involves the deterioration of fish diet. People with a high level of Tau excretions in 24U showed a preferable result in FBG, TC and non-HDL-C compared to those with a low level of it. The fact that administration of omega-3 fatty acids including DHA and EPA, rich in fish products, was effective to dyslipidemia is consistent with our present result that the high Tau group showed elevated DHA and suppressed TC and non-HDL-C level [19]. Changes in dietary habit to eat less fish might accelerate the increasing MS risk factors. It was reported that Tau excretion in 24U showed a significant inverse correlation with ischemic heart disease in men and women and a positive correlation between fish intake and CVD in Japanese [6]. In the present study among the high Na excretion group, the middle-aged with high Tau excretion showed a lower percentage of MS risks, especially based on increased BP, than young adults with low Tau excretion (data not shown). The beneficial effect of Tau was supported by the finding that Tau supplementation showed cardio-protective effects in stroke prone spontaneously hypertensive rats (SPSHR) given high salt diets [20]. The correlation between age and salt intake was detected in the present study; however the middle-aged rather than young adults would be somewhat protected by Tau from the adverse effect of salt on increasing MS risks.

 In the past, Lake Victoria provided a wide variety of fish species as well as important subsistence fishing. The people living nearby benefitted from the valuable nutrition supply of fish in the lake. After the introduction of exogenous fishes like Nile perch to the lake, local small-scale fishing was pushed aside by large-scale commercial fishing which caused small fish, the main source of nutrition, to be unavailable for inhabitants [21]. The change of fishery, biology, environment and socio-economics might bring alteration in food custom, which would have an impact on health conditions in the long view.

## Conclusions

Young adults in Mwanza have a tendency to eat less fish products than middle-aged as indicated by Tau excretion in 24U and omega-3 fatty acid in plasma. The changes in food habit including the low rate of fish intake especially in young men will increase MS risks, such as hypertension.

## List of abbreviation used

AI: atherosclerosis index; BMI: body mass index; BP: blood pressure; CARDIAC Study: Cardiovascular Diseases Alimentary Comparison Study; CVD: cardiovascular diseases; DBP: diastolic blood pressure; DHA: docosahexaenoic acid; EPA: eicosapentaenoic acid; FBG: fasting blood glucose; HbA1c: haemoglobin A1c; HDL-C: high-density lipoprotein cholesterol; MS: metabolic syndrome; Na/K: sodium-potassium ratio; non-HDL-C: non-high-density lipoprotein cholesterol; SBP: systolic blood pressure; TG: triglycerides; WC: waist circumference.

## Competing interests

The author(s) declare that they have no competing interests.

## Authors' contributions

YY designed the study. MM and AM collected the data under assistance by HM and MN. ZM helped the operation and JM coordinated the project. AH analyzed the data and drafted the manuscript. All authors commented and approved the final version of the paper.
